# North-South Corridor Demonstration Project: Ethical and Logistical Challenges in the Design of a Demonstration Study of Early Antiretroviral Treatment for Long Distance Truck Drivers along a Transport Corridor through South Africa, Zimbabwe, and Zambia

**DOI:** 10.1155/2013/190190

**Published:** 2013-03-31

**Authors:** G. B. Gomez, W. D. F. Venter, J. M. A. Lange, H. Rees, C. Hankins

**Affiliations:** ^1^Department of Global Health, Academic Medical Center, University of Amsterdam & Amsterdam Institute for Global Health and Development, Trinity Buildings, Building C, Pietersbergweg 17, P.O. Box 22700, 1100 DE Amsterdam, The Netherlands; ^2^Wits Reproductive Health and HIV Institute, The University of the Witwatersrand, South Africa

## Abstract

*Background*. Long-distance truck drivers are at risk of acquiring and transmitting HIV and have suboptimal access to care. New HIV prevention strategies using antiretroviral drugs to reduce transmission risk (early antiretroviral therapy (ART) at CD4 count >350 cells/**μ**L) have shown efficacy in clinical trials. Demonstration projects are needed to evaluate “real world” programme effectiveness. We present the protocol for a demonstration study to evaluate the feasibility, acceptability, and cost of an early ART intervention for HIV-positive truck drivers along a transport corridor across South Africa, Zimbabwe, and Zambia, as part of an enhanced strategy to improve treatment adherence and retention in care. *Methods and Analysis*. This demonstration study would follow an observational cohort of truck drivers receiving early treatment. Our mixed methods approach includes quantitative, qualitative, and economic analyses. Key ethical and logistical issues are discussed (i.e., choice of drug regimen, recruitment of participants, and monitoring of adherence, behavioural changes, and adverse events). *Conclusion*. Questions specific to the design of tailored early ART programmes are amenable to operational research approaches but present substantial ethical and logistical challenges. Addressing these in demonstration projects can inform policy decisions regarding strategies to reduce health inequalities in access to HIV prevention and treatment programmes.

## 1. Introduction

Recent studies have shown the impact of population mobility and transport infrastructure on the spread of HIV in Africa [1, 2]. While mobility can be considered an underlying determinant of HIV transmission, proximal factors include low perceptions of risk, differential HIV prevalence between the areas of origin and destination, and overlapping sexual networks [3]. Long-distance truck drivers, in particular, are highly mobile, spending long periods on the road away from their families. Their mobility in and of itself does not increase their risk of HIV acquisition or transmission directly. However, separation from families and communities, loneliness, and lack of access to HIV prevention, treatment, and care services make this mobile population particularly vulnerable to HIV, potentially favouring the spread of HIV into other lower-risk sexual networks [4–8]. 

Among truck drivers in the region, HIV prevalence estimates from the literature are scarce and highly heterogeneous (from 5% to over 50%): in 1993, 15% of drivers tested positive for HIV in Cameroon [9]; in 1994 and 1995, two studies in Kenya showed a 26% and 27% prevalence of HIV among truck drivers and their assistants attending roadside clinics at the Athi River and in Mariakani, respectively [10, 11]; from 2001 to 2007, HIV prevalence did not change significantly in truck drivers in Guinea (7.0% and 5.3%, resp.) [12]; in 2002, a survey of truck drivers visiting sex workers at truck stops in KwaZulu Natal found an overall prevalence of 56% [13]; and, in 2011, HIV infection in long-distance truck drivers in the Niger Delta of Nigeria was estimated at 10% [4, 14]. A 2004 modelling study estimated new HIV infections at 3000–4000 annually among sex workers and their clients (mainly truck drivers) on the major transport corridor between the port of Mombasa, Kenya, and Kampala, Uganda [15–17]. This corresponded to a high incidence estimate between 0.66 and 0.89 per 100 person/years in truck driver clients of sex workers, assuming HIV prevalence in truck drivers to be 20%, condom use frequency to be constant (78% for contacts with sex workers), and HIV prevalence in sex workers to vary between 30% and 50%.

**Figure 1:**
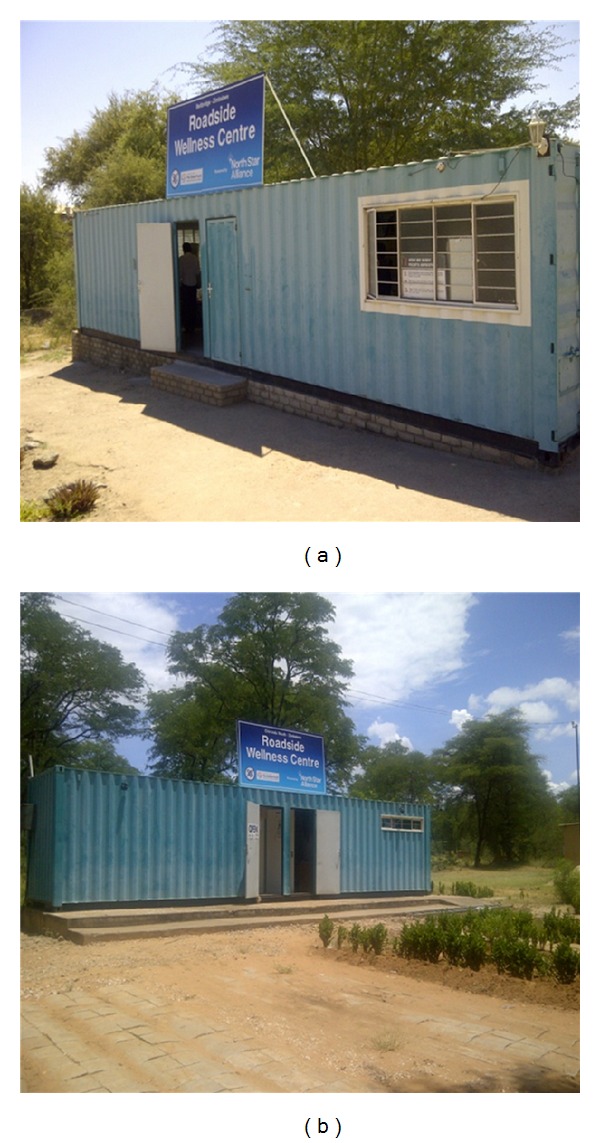
Roadside Wellness Centres in Chirundu South and Beitbridge, Zimbabwe.

In order to provide suitable prevention and treatment services to truck drivers and other vulnerable populations along transport corridors, several initiatives have been funded in southern Africa [18]. Notably, the Walvis Bay Corridor Group in Namibia aims to facilitate the development and implementation of comprehensive HIV prevention workplace programmes [19]. In Zambia, one initiative funded by USAID and run by Family Health International (FHI), “Corridors of Hope,” aims to reduce the transmission of HIV by delivering healthcare services to high-prevalence border and transportation communities. It supplements referral activities with education and economic improvement activities and community outreach [20]. In 2006, FHI published an evaluation reporting the services to be acceptable and rated as high quality by clients. A trend in positive behavioural change and an increase in uptake of services were recorded [21]. This initiative has secured funding until 2014 [22]. Another initiative in the region is the North Star Alliance, a public-private partnership promoting a corridor-based approach to healthcare delivery through Roadside Wellness Centres (RWCs) opened and managed along major traffic corridors [23]. North Star Alliance's RWCs are semimobile converted shipping containers registered with national Ministries of Health as centres authorised for the provision of prevention services consisting of primary care and referral to other primary care centres and hospitals. RWCs are located in hotspots along the transport corridors and offer HIV prevention services including HIV testing and counselling (HTC), condom distribution, and syndromic treatment of sexually transmitted infections (STIs), as well as other basic primary care services, to mobile workers and the surrounding communities. In 2011, North Star Alliance expanded its network to a total of 22 RWCs in east and southern Africa. These wellness centres are connected through an information technology system (COMETS—Corridor Medical Transfer System) serving as an electronic health passport. The system uses a unique personal identification number and is resilient to common operational challenges such as power outages and breaks in connectivity (see Figure 1). 

**Figure 2:**
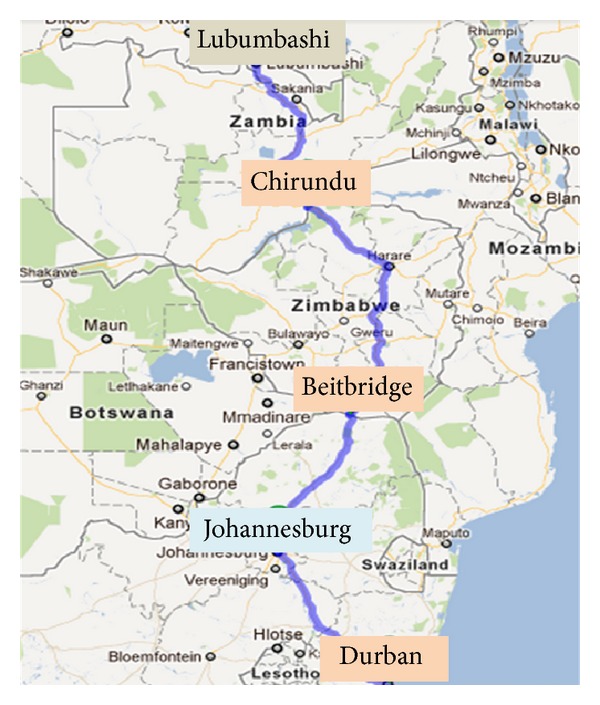
North-South corridor across South Africa, Zimbabwe, and Zambia.

In April 2012, North Star Alliance was awarded a programme grant from the Dutch Ministry of Foreign Affairs (regional office for southern Africa) to expand its services to link HIV-positive patients to national care and provide ART monitoring services as part of a basic healthcare package in the busiest corridor of southern Africa: the “North-South” transport corridor across South Africa, Zimbabwe, and Zambia (Figure 2) [18]. Current RWCs along this corridor are located in Durban (South Africa), Beitbridge and Chirundu South (Zimbabwe), and Chirundu North (Zambia). In 2012, 35% of all services provided were HTC sessions. Of these, sessions for truck drivers represented almost a third of the total HTC sessions provided in these four RWCs (personal communication, North Star Alliance, January 2013). These truck drivers tend to cover long distances and are not all members of any particular union. The ART service expansion in this corridor is expected to be established in 2013. Should this service expansion prove successful, a demonstration project for early ART for truck drivers who do not meet national treatment eligibility criteria would be feasible. Demonstrating that early ART can be safely and effectively delivered to truck drivers will increase the probability that this mobile population will be among the first to benefit from future anticipated changes in ART eligibility in South Africa, Zambia, and Zimbabwe.

Recently, early ART initiation has been shown to lead to a 96% reduction in virologically linked HIV transmission to HIV-uninfected partners when comparing treatment initiation at CD4 counts between 350 and 550 cells/*μ*L to a delayed start at CD4 counts below 250 cells/*μ*L [24]. Furthermore, in recently published documents, WHO has provided guidance on the implementation and evaluation of demonstration projects of preexposure prophylaxis and early initiation of treatment [25, 26]. Several conditions are prerequisites for effective engagement of patients across the spectrum of HIV care services [27, 28] for these new uses of antiretrovirals for prevention to realise their promise. First, HIV testing should be widely available to facilitate early identification of HIV-positive patients. The identified individuals should be willing and able to start ART at high CD4 counts. HIV-positive patients on ART should then be carefully monitored and supported to maintain high levels of adherence and achieve viral suppression, rendering them less infectious [29] and ultimately decreasing HIV transmission [27, 30, 31]. Mathematical models have assessed the promise of an early ART approach in the general population, with mixed results [32–37]: although similar reductions in incidence are estimated for the short term, long-term predictions vary significantly [38]. However, recent modelling including mobility and limited retention in care have shown that structural barriers to care could be an important limitation for these programmes [39].

In this paper, we present the protocol for a demonstration study to evaluate the feasibility, acceptability, and cost of an early ART intervention for truck drivers as part of an enhanced strategy to improve adherence and retention in care. This occupational group presents specific ethical and logistical challenges that need to be considered in the design of treatment services and expansion to include early initiation of ART. We aim to discuss these challenges in order to inform the development of ongoing and future research that focuses on translating trial results into programme effectiveness in operational settings [27, 30, 40, 41]. The strength of this project lies in combining research findings with routinely collected data in an economic model that would provide feedback to health service programme providers. In particular, reliable cost-effectiveness data would thus inform the development of future HIV prevention and treatment strategies. 

## 2. Materials and Methods

### 2.1. Aim and Objectives

The aim of this study is to investigate whether an early start of ART (CD4 count > 350 cells/*μ*L, i.e., before meeting national eligibility criteria) combined with an enhanced HIV care and prevention package would represent a feasible and affordable strategy for mobile populations. The primary outcome will be the number and proportion of patients offered early ART who accept ART and are retained in care with plasma HIV-1 RNA load of less than 400 copies/*μ*L one year after treatment initiation. Viral load is a good predictor of adherence and HIV transmission probability. Participants who do not achieve this outcome would be classed as virologic failures.

Secondary outcomes include

- individual CD4 counts at diagnosis,
- uptake of early ART (proportion of those who are offered early ART who accept and start early ART),
- reasons for nonacceptance of early ART,
- retention in the programme (proportion of those who accept early ART that continue on ART at each follow-up visit),
- self-reported adherence to treatment,
- adherence measured through u-boxes that electronically record the timing of doses,
- reasons for dropout and/or nonadherence to the early ART programme (including mortality, transfer to nonstudy clinics, and loss to followup without identifiable cause),
- self-reported sexual risk behaviours (e.g., unprotected sex in the past month),
- kidney function at baseline and followup (i.e., creatinine clearance),
- cost per unit of output (e.g., cost per patient on ART as per national guidelines, cost per patient starting early ART).

### 2.2. Study Population, Sample Size, and Sampling

Long distance truck drivers and their assistants would be invited to participate if they are HIV-1 positive with a CD4 count between 350 and 500 cells/*μ*L, are ART naive, and present no contraindications. These participants are not eligible for treatment under current national guidelines in the three countries through which the corridor runs. In all countries, ART is provided free of charge, provided the following eligibility criteria are met. In South Africa, HIV-positive patients are eligible for treatment if their CD4 count is <350 cells/*μ*L or irrespective of CD4 count for those presenting at a WHO stage IV [42]. In Zimbabwe and Zambia, ART is recommended for all patients presenting with WHO stage III or IV irrespective of CD4 count or at CD4 count <350 cell/*μ*L [43, 44]. In addition, in Zambia, all HIV-positive persons within a serodiscordant couple are recommended to start ART irrespective of CD4 count [43]. We would restrict eligibility to those with a CD4 count <500 cell/*μ*L in anticipation of changes in WHO normative guidance in 2013.

The sample size to detect a viral suppression rate at 12 months of 75%  ± 7% with 80% power would be 150 truck drivers and their assistants. We would approach a total of 200 for participation, expecting 15% refusal and a further 10% rate of loss to followup. To our knowledge, there are no studies documenting retention in HIV care or viral suppression rates among truck drivers. Thus, the assumption of a 75% viral suppression rate is based on the rates observed in a Kenyan m-health randomised controlled trial [45], assuming a higher rate due to the use of a single pill regimen. In this trial, the authors observed a viral suppression rate of 57% in the intervention group and 48% in the control group. The intervention included periodic interactive text messages and reminders to those patients receiving ART in three different HIV clinics representing regional diversity in health settings. All patients were adults starting ART as per national guidelines in routine service. 

**Figure 3:**
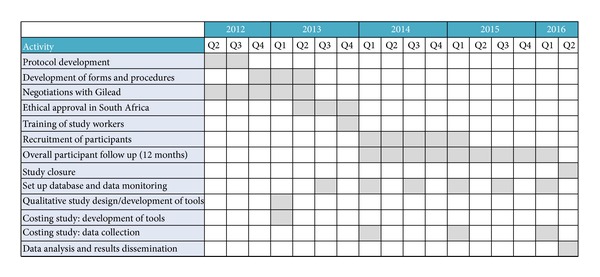
Project timeline detailing related activities.

We estimate that recruitment would be slow. During the six months before the start of the study, we would monitor the client flow in the RWCs to estimate how many would be eligible for enrolment. If a total of 100 truck drivers access HTC per week at the four RWCs, we would require 15 months of screening (*n* = 6, 175) to identify the necessary sample of eligible patients. This is assuming an HIV prevalence of 13% (*n* = 803) (personal communication, North Star Alliance, January 2013). This prevalence estimate is a conservative estimation based on routine data collected by North Star Alliance and it is significantly lower than the prevalence reported in the literature for South Africa [7]. Of these HIV-positive clients, two-thirds would be eligible for treatment as per guidelines (*n* = 535 patients). Of the 268 patients left, three quarters (*n* = 201) would be eligible for early treatment [46] and approached to participate. If the first participants were to be recruited during the third quarter of 2013, the project timeline detailing related activities would resemble Figure 3.

### 2.3. Study Design and Procedure

We would follow this observational cohort of truck drivers through the care pathway for early treatment and use a mixed methods approach, including quantitative, qualitative, and economic analyses. 

#### 2.3.1. Demonstration Study

Participants would be recruited at an ART initiation site in Alberton (Johannesburg, South Africa). In this location, an RWC providing HTC would be linked to an ART initiation site where patients found to be HIV positive would have access to further testing and evaluation for ART initiation. Eligible patients (HIV-1 positive, ART naive, and CD4 counts between 350 and 500 cells/*μ*L) would be counselled at the initiation site after receiving results of CD4 counts about the potential benefits and risks of starting ART earlier. Patients consenting to participate would then be assessed by a physician. Patients presenting a history of pathologic fracture or risk factors for bone loss, creatinine clearance level below 70 mL/min, or an elevation of alanine aminotransferase (ALT) levels at screening would not be included in the study. For eligible participants, and to avoid high early dropout, we plan a counselling run-in period where truck drivers are given information and asked to consider early ART before enrolment. At a second visit, a month later, they would be started on early ART. The drug of choice for this pilot is QUAD (elvitegravir 150 mg/cobicistat 150 mg/emtricitabine 200 mg/tenofovir disoproxil fumarate 300 mg) which is a once-a-day fixed dose combination (FDC) regimen recently approved by the U.S. Food and Drug Administration [47] and for which registration is likely to be sought in South Africa in 2013. Participants would be counselled about the common adverse drug reactions observed in previous clinical studies (i.e., nausea, diarrhoea, abnormal dreams, headache, and fatigue). They would be advised to contact a specific telephone number if they have any concerns about these adverse drug reactions—this number will be free of charge from South Africa, Zimbabwe, and Zambia. Participants will be provided with a one-month supply of drugs and a u-box. They will be asked to provide contact details including mobile phone numbers. At recruitment, the numbers provided would be verified by calling the participant and any preferences for a primary number recorded. We would also ask for a personal contact in case the clinic has difficulty reaching them to notify them of changes in the study or to followup on missed appointments. Patients would be asked to consent to home visits (one month after missing a follow-up visit) in the instance that we are unable to track them through phone contact or their personal contact. The latter would only be used after three attempts to reach the truck driver's own mobile phone. Participants would be informed that SMS will be sent to them with reminders of appointments and pertinent information about adherence (e.g., reminders of pill supplies running low and weekly reminders of the importance of adherence to the treatment regimen).

Patients would be asked to come back for followup every month for a three-month period, and then every two months for the next nine months. This follow-up schedule is in agreement with local guidelines for the first three months of treatment. After the initial three months, national guidelines advise biannual followup [42–44]. Follow-up sessions would be scheduled at the initiation site of Alberton, Johannesburg in South Africa. It is expected that only truck drivers working in the route through Johannesburg would be able to access this service, which may slow the recruitment process. Nevertheless, we would train the staff at all RWCs along the corridor to be able to advise in case of adverse reactions and to update any appointment that might need to be rescheduled. During follow-up visits, participants would be provided with drug refills. The following tests would be done at every follow-up visit: urine glucose, urine protein, creatinine clearance, ALT, CD4 counts, and plasma viral load. At baseline and then every three months, the study team plans to administer a sexual behaviour and adherence questionnaire. Treatment would be discontinued if creatinine clearance falls below 50 *μ*L/min. Treatment failures are defined as two consecutive plasma viral loads over 400 copies/*μ*L. We will investigate all treatment failures for the presence of drug resistance. To our knowledge, there are no data available on resistance emergence among this population. We expect these data to be an important secondary outcome of our project. All patients discontinuing (due to side effects or for other reasons) would be assessed by a physician and a new treatment regimen prescribed according to national guidelines. We will discuss relevant local health services before the commencement of the study to ensure ongoing treatment for demonstration study participants at North Star Alliance sites, as it is expected that their CD4 count will be above national treatment guidelines at the end of the study. 

It is expected that Gilead Sciences together with a local generic partner will apply for regulatory approval and licensure of QUAD in South Africa during 2013. If approved, at the end of the study period, patients would be discharged to the national health system. If approval is still pending at the end of the study, Gilead Sciences will be asked to support the continuation of patients on the study protocol drug until approval is received and discharge is possible. This is a key point for discussion with Gilead Sciences, the national regulatory authority, and the local ethics committees before study initiation. In the absence of a negotiated agreement, the demonstration project would not proceed. 

#### 2.3.2. Qualitative Study

In order to provide in-depth information on the acceptability of the early ART approach and changes in adherence during use, we would randomly select 10% of the participants (*n* = 20) who would be asked to undergo three in-depth interviews at recruitment and during two follow-up visits (i.e., at months 0, 6, and 12). In addition we would conduct an in-depth interview with any participant choosing to discontinue ART or choosing to withdraw from the study at the moment the patient decides to withdraw and after consent has been given.

#### 2.3.3. Costing Study

If the study goes forward, we will undertake a costing exercise to explore the feasibility of early ART in order to inform future resource allocation within North Star Alliance. The evaluation will be done from a provider's perspective—only healthcare provider costs will be included—extra costs for the patients (in terms of transport, time off work, etc.) will not be included. Data will be collected through observations at the site, interviews with the healthcare workers, and review of financial statements in the clinic (such as utility bills) over the duration of the study. We will include capital costs (equipment, buildings, nonrecurrent training, outreach efforts, and start-up activities), as well as recurrent costs (personnel (all types), supplies, operations, and maintenance of buildings) [48]. Particular attention will be paid to quantifying the time requirements related to early ART initiation activities (e.g., counselling and extra laboratory testing during monitoring visits related to early ART initiation) and to separating monitoring and care activities from research-related activities (e.g., sexual behaviour questionnaires) in order to help inform subsequent human resource needs (e.g., staff time and possible task shifting opportunities) should the intervention be considered for scale-up.

### 2.4. Data Analysis and Dissemination

**Figure 4:**
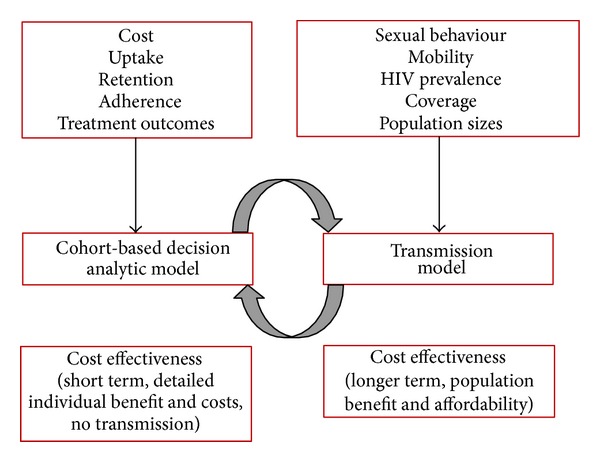
Schematic representation of the model-based evaluation.

We plan a model-based evaluation of the cost effectiveness of early ART initiation (Figure 4). The evaluation will be in two phases—first a cohort-based model will be developed to explore the short-term and individual-level effects of this intervention as well as the costs to the healthcare provider by funding source. Data from the surveys and biological data from the follow-up visits will be used to inform the effectiveness evaluation. Data from the costing study will inform the cost evaluation. Data from the qualitative interviews will be analysed to explain reductions in adherence and programme retention. Long-term projections and evaluation of the broader population-level effects will be estimated through a transmission model. This model will focus on truck drivers and their partners and will require data from sources external to the demonstration study, such as relative population sizes, sexual behaviour information, and HIV prevalence in sex workers. We plan to source these data from ongoing studies and the literature. We would present both the study protocol and the results at national and regional meetings with policy and decision makers before commencement of and during the study, as well as after data analysis. The findings of the economic modelling will be communicated to North Star Alliance and to the National Bargaining Council for the Road Freight Industry in which both employers and the trade unions are presented, as well as to all stakeholders in this study through reports and presentations. The findings will be formally presented to the National Department of Health and to the South African National AIDS Council. 

## 3. Ethical and Logistical Considerations 

This study has been designed following local and international good clinical practice [49, 50] and according to Good Participatory Practice guidelines [51]. Input from representatives of the truckers industry including both employers and unions was obtained in the development of services for the North Star Alliance. An advisory committee, including members of the trucking industry, would be formed to provide input into study design and advice during study conduct. The protocol presented here will be reviewed by the relevant South African ethics committee before study implementation and by the South African Medicines Control Council. In this section, we discuss three main ethical and logistical considerations examined during the protocol development.

### 3.1. Drug of Choice

Current treatment regimens considered in the guidelines of South Africa, the country where the participants will be started on early ART, may produce side effects of variable intensity and duration in a sizable proportion of people at treatment initiation. In particular, it is recommended that patients starting efavirenz avoid driving due to sleepiness and that patients starting on nevirapine seek immediate medical care in the event that they develop drug-related rashes that are potentially life threatening. Furthermore, nevirapine is not recommended for patients with higher CD4 cell counts because the risk of severe rashes is higher. These conditions make these two first line drugs unsuitable for a population of truck drivers who are unable to take leave of absence from their work during treatment initiation. The drug of choice for this pilot is QUAD (elvitegravir 150 mg/cobicistat 150 mg/emtricitabine 200 mg/tenofovir disoproxil fumarate 300 mg). Specifically, it was selected because (1) it is a once-a-day single pill regimen and (2) has shown mild side effects in trials (i.e., nausea, diarrhoea, abnormal dreams, headache, and fatigue), and (3) it does not produce drowsiness.

This choice presents several challenges. QUAD contains two previously approved HIV drugs plus two new drugs: elvitegravir and cobicistat. Following safety and efficacy results in two double-blind clinical trials [52, 53] of 1,408 ART-naive adult patients, QUAD received FDA approval recently [47]. While Gilead Sciences (the manufacturer) is required to conduct additional studies to help further characterise the drug's safety in women and children, we propose its use only in men and to follow QUAD's label advice to health care providers on how to monitor patients for kidney or bone side effects. However, an application to South African authorities for approval of this drug combination has not been filed yet. Gilead Sciences is expected to submit the application to the South African authorities for regulatory approval in the near future. As application for licensure is likely to be ongoing for the duration of this study, the MCC will be approached for permission to conduct this trial as an observational study conducted to GCP and GPP standards, with patients being started on QUAD only in South Africa and with no drug distribution in the other countries. 

A second issue is the current cost of this regimen. The drug will be available free of charge during the study. However, at an estimated US$28,500 in the USA [54], it would not be accessible to our population in the South African setting once the study finishes, even were it to receive regulatory approval. However, there are ongoing discussions to produce a generic tablet in South Africa, which will help ensure its availability in the longer term. 

### 3.2. Recruitment of Patients into the Study and Treatment Initiation

Although trained nurses are currently allowed to initiate ART and in the future would potentially be able to initiate early ART (patients at higher CD4 counts generally present fewer clinical complications), a physician will assess and initiate early ART in this demonstration study. The model for ARV treatment expansion within this corridor is one that sees physicians initiating ART and nurses monitoring followup. Our analysis will explore the potential savings of task shifting from this model to a nurse-led early ART programme. Because during the study, participants may have difficulties separating clinical care from research since both ART as per guidelines and early ART will be physician-initiated and nurse-monitored, the difference will be reinforced in the informed consent process.

A signed informed consent from patients would be obtained for recruitment to the cohort and follow-up procedures. Information would be made available during the consent procedure about the implications of participating in the study—including the risks, consequences, and benefits of starting treatment early. Detailed clinical and counselling materials as well as consent forms would be available in local language explaining the possible individual-level benefits and potential risks, as well as the possible public health benefits. During the informed consent process, we would define alternative strategies to start ART and how to access ART services as part of the RWC services and through other sites. It would be made clear that all patients will benefit from access to pre- and postcounselling as well as to standard of care services during their pre-ART phase should they refuse to participate. All patients would also have access to the prevention services available (condom promotion and distribution, STI treatment, partner disclosure advice, and counselling) independently of their participation status. 

In relation to the cost study, we plan to ask all healthcare workers to consent to observations of practice and interviews. Data from the observations would not be shared with the management of the clinic and the study team would ensure that staff are aware that there would be no consequence to their job situation should they choose not to participate in the study. 

### 3.3. Monitoring: Adverse Events, Behavioural Change, and Adherence

Each participant would be followed using a case report form that includes clinical information at recruitment and follow-up visits. The study team plans to collect and report adverse events in these forms. Study participants would be provided instructions for contacting the study site to report any untoward medical occurrences they may experience, except for possible life-threatening events, for which they would be instructed to seek immediate emergency care. Where feasible and medically appropriate, participants would be encouraged to seek medical care where the study physician is based. Standard operating procedures would be developed for quality assurance, describing most common adverse events (i.e., rashes). With appropriate permission of the participant, whenever possible, records from all nonstudy medical providers related to adverse events would be obtained and required data elements recorded on study case report forms. All participants experiencing an adverse event would be followed clinically, until it resolves, returns to baseline, or stabilises. Information on Grade 3 and higher adverse events would be reported to the Medicines Control Council as well as the relevant ethics committee. All participants would be covered by comprehensive insurance for study-related injury and damage. The study would identify a laboratory that is accredited by local and international agencies for the conduct of HIV research of this nature. 

Behavioural change toward more risky behavior after initiation of ART would be assessed through data collection on risk behaviours at follow-up visits. During these visits, we would reinforce counselling to reduce risk behaviours. Adherence to the drug regimen would also be monitored using the u-boxes, with both adherence and retention promoted in the programme using mobile phones (i.e., SMS reminders of appointments and counselling advice). Adherence and retention would be closely monitored during the first three months of the study. 

Finally, South Africa has recently approved its National Strategic Plan for HIV, TB, and STIs for 2012–2016 [55]. Based on the local “Know your Epidemic” data, certain key populations have been identified as being central to the HIV prevention effort. Included in these key populations are truck drivers and sex workers. Interventions that explore novel approaches to lower HIV risk in these populations support South Africa's efforts to reduce new HIV infections by 50% by 2016. The South African National AIDS Council (SANAC), chaired by the Deputy President, is coordinating these efforts and this protocol would be shared with SANAC before implementation. 

## 4. Conclusion

Prevention interventions are a national priority for key populations in South Africa, and research for innovation in this field is a national priority. Many questions specific to early ART initiation, whether in the general population or in transport workers, are amenable to operations and health systems research. The use of a locally unregistered drug increases the regulatory and ethical requirements of the study. Nevertheless, it is important to address key operational, regulatory, and ethical aspects of providing early ART services to a key population. When deciding on prevention interventions, prioritisation has to occur based on cost effectiveness, feasibility, and acceptability. As resources are limited, prioritisation must also take into account fairness and equity, particularly when vulnerable groups are being considered. Further, while engagement of truck drivers in an early ART programme has the potential to benefit both the individual (increasing access and retention in care for a mobile population) and the population (decreasing transmission to either lower-risk or higher-risk networks), there is an important need to address stigma and avoid discrimination and any characterisation of this population as a “vector for HIV transmission,” thereby exacerbating already existing health inequalities.
